# A Mixed-Methods Analysis and Personal Narratives of Black Maternal Health Experiences in the American Healthcare System

**DOI:** 10.7759/cureus.81926

**Published:** 2025-04-08

**Authors:** Micah E Swaby, Genevieve Anyimadu, Modjadji Choshi, Prajakta Belsare

**Affiliations:** 1 Biology, James Madison University, Harrisonburg, USA; 2 Research, Eastern Virginia Medical School, Norfolk, USA; 3 Nursing, James Madison University, Harrisonburg, USA; 4 Statistics, James Madison University, Harrisonburg, USA

**Keywords:** advocacy, black maternal health, healthcare disparities, healthcare education, maternal mortality

## Abstract

Purpose

The growing disparities and vulnerabilities in healthcare have contributed to alarmingly high maternal mortality rates for Black/African American women. In the United States, the maternal mortality rate is exponentially higher than that of other industrialized nations. Furthermore, within this nation, Black women face a disproportionately greater risk of maternal death. Therefore, blending the knowledge from these realities, it is evident that the immense number of women dying in our industrialized nation, compared to others, are primarily Black women. With knowledge of this devastating disparity, the fundamental objective of this project is to provide a voice for the Black/African American individuals whose lives are claimed prematurely due to systemic health disparities and lack of quality care. Our study aims to learn more intimate and personal recollections of the birthing experiences of Black/African American women as they are currently the most vulnerable.

Method

From June to December 2023, a mixed-methods study was conducted that included a quantitative questionnaire, which received 60 responses. Fourteen of those participants also took part in the qualitative portion, consisting of four interview groups that provided more insights into their experiences.

Results

This paper presents the mixed-method findings from both the questionnaire and interview groups, which revealed a spectrum of experiences. The survey results were analyzed and categorized as either sufficient care or insufficient care necessitating improvement. Additionally, participants provided intimate details through written and verbal responses, which have been illuminating.

Conclusion

Ultimately, prioritizing the voices of Black women through a patient-centered approach to addressing maternal mortality highlights the need for culturally appropriate interventions, enhanced healthcare education, and strengthened advocacy. These efforts are essential in creating a more equitable system that improves maternal health outcomes and reduces racial inequities.

## Introduction

In the United States, the stark disparities in maternal health outcomes, particularly those affecting Black women, have gained increasing attention as their experiences highlight systemic barriers and inequities in healthcare. Throughout the COVID-19 pandemic, health disparities have worsened for Black women with the population experiencing the most substantial increase in poor maternity outcomes [1].

Objectives

This study embarks on a comprehensive exploration of Black maternal health with an aim to amplify the voices and stories of Black women who have experienced childbirth in the United States. By investigating the multifaceted factors contributing to healthcare disparities, the ultimate objective of this study is to increase public awareness surrounding the maternal health crisis, educate healthcare professionals and policymakers, and advocate for systemic interventions that can save lives and improve patient outcomes.

Background

The foundation of this project began with an in-depth analysis of existing literature and recent studies. This exploration uncovered a wealth of critical information, with several key findings shaping the direction of our work. Most remarkably, the research conducted at the Centers for Disease Control and Prevention found that over four out of every five pregnancy-related deaths are preventable [2]. Hence, what should we be doing differently to prevent the loss of lives unnecessarily? What immediate actions can be implemented? What systemic changes are needed to address this crisis? In addition, another source highlighted that healthcare professionals consider Black Americans to be less cooperative, less compliant, and less responsible [3]. Rooted in historical prejudice, this conscious and unconscious bias can have fatal consequences for Black Americans navigating our healthcare system. Not only does bias influence the quality of treatment individuals receive, but it has also fostered widespread distrust and fear, leading countless Black Americans to avoid seeking medical treatment altogether. Hence, what can we do in order to eliminate bias and ensure fair treatment for every human being? Furthermore, another distinctive theme is the proactive steps that Black Americans are taking to confront healthcare inequities. Many are turning to intimate, community-based health systems as alternatives to larger, often impersonal medical institutions. This shift highlights the resilience of communities as they support one another and advocate for justice to improve health outcomes.

With these research findings in mind, we still had lingering questions. If the quantitative data show that Black women are dying at a significantly higher rate than their White counterparts, then how can we listen and learn more about the personal experiences of Black individuals? Our study seeks to amplify these narratives to foster understanding, drive meaningful policy changes, and contribute to a more equitable healthcare system.

## Materials and methods

Study timeline

Data collection for this study was conducted over a six-month period from June 2023 to December 2023. From June to November, we focused on participant recruitment and the collection of survey responses. In late November, participants were contacted to inquire about their interest and availability for the focus groups. The focus group discussions were conducted virtually over one weekend in December. Following the completion of these sessions, data collection officially concluded, and we transitioned to the analysis phase.

Subject recruitment**

Our project consists of a mixed-methods study utilizing a questionnaire (quantitative) and focus group interviews (qualitative). To initiate research on human participants, ethical considerations and precautions were followed. Prior to commencing data collection, approval from the Institutional Review Board (IRB) was obtained (protocol #23-4129) to ensure compliance with ethical standards and the rights of our participants. Recruitment efforts targeted diverse community groups with an aim of socioeconomic and geographic representation. Eligible participants were Black/African American women aged 18 and above who had been pregnant or given birth in an American hospital. We aimed to establish clear inclusion and exclusion criteria. Inclusion criteria encompassed adult Black or African American individuals whose sex assigned at birth is female and who were either pregnant, have been pregnant, or have given birth in the United States where they currently reside. Conversely, our exclusion criteria included individuals under 18, those who were institutionalized, and those unable to fully provide consent.

Our research aimed to cover a range of geographic locations primarily along the East Coast of the United States, including urban, suburban, and rural areas, with the intent of gathering a range of experiences and viewpoints to be represented in our study sample. To reach eligible participants, recruitment strategies involved social media, word of mouth, and connections within our community in efforts to distribute our survey flyers to maximize participant involvement. We made flyers that concisely described the purpose of our study, the eligibility for participation, and our contact information for individuals interested in learning more. To engage directly with the Black communities around us, we actively explored people throughout our campus at James Madison University and became involved in the Ole School Alumni Scholarship Group (OSASG) while connecting with Sisters in Session (a group at JMU that supports Black/African American female faculty), church groups, and many other Black organizations that were willing to help. We aimed to establish rapport in these spaces essential for facilitating meaningful participation in our research endeavors. 

Procedure

Data collection was initially conducted through JMU QuestionPro, an online survey platform, and then switched to JMU Qualtrics to maximize security efforts. For the very few participants who had already completed the survey on QuestionPro, their responses were manually transferred into Qualtrics without any alterations. The survey template was carefully adapted from an existing template used in Norway to assess patient perspectives surrounding their maternal care [4]. To better fit the objective of this project, the survey was adjusted to reflect the context of Black maternal health in the United States, encompassing a broad array of questions about demographic information, pregnancy, birth history, access to healthcare, experiences of discrimination, and overall maternal well-being. The modification process entailed thoughtful consideration of cultural nuances and factors that would provide relevant application of the survey to our study population. The survey questions can be found in the Appendix (Table 2). 

Subsequently, focus groups were conducted to further understand the individual experiences that Black women faced during and after pregnancy. We prioritized the protection of participant confidentiality and privacy by ensuring that all identifiable information, including names, medical records, and health providers, were kept confidential outside the review process. All records, including audio recordings, were kept secure in a locked location. Participants were informed about the benefits, risks, and the confidential nature of the focus group interviews, in which their contributions would be aggregated and reported in a manner that protects their anonymity. To lead the focus groups in a step-by-step manner, open-ended questions were developed to explore their story from the beginning of their healthcare history through the depth of their experiences giving childbirth. Furthermore, questions were asked regarding their social determinants of health (i.e. health insurance, safety and living conditions, support, etc.). In the end, participants were invited to share what they think could be better to help women from experiencing any negative qualities of birth. The interview guide was adapted using a guide from the CDC [5]. The outline of questions can be found in the Appendix (Table 3).

Quantitative analysis

For our research study, all of the quantitative data gathered through the surveys were collected using the Qualtrics platform. To better analyze the data, we transferred all of the information into Microsoft Excel. The purpose of our quantitative analysis was not to test a hypothesis or establish statistical significance, but rather to illustrate the range of perspectives and lived experiences of our participants. By presenting their responses numerically, our goal was to provide readers with a broader, more nuanced understanding of their stories, encounters, and perspectives. 

For each question, we analyzed the data to see how our participants generally answered by dividing them into binary categories based on a “sufficient” or “insufficient” response. The majority of our questions fell on a five-point scale ranging from poor to excellent. If participants chose an answer that equated to a four or five, we considered their answer as sufficient as that signifies their care went well. If participants chose a three or less, we considered their answer as insufficient as it highlights that there are improvements that can be made. If participants did not answer or selected “N/A” as their response, they received a zero on our scale and were removed from the analysis for that question.

Qualitative analysis

The qualitative data for this study comprise in-depth audio recordings of four focus groups, each lasting approximately an hour. These sessions were meticulously transcribed, and all identifying information of participants, including names of individuals and their providers, were anonymized. Following the interview groups, the data were transcribed and thoroughly analyzed to identify recurring themes and commonalities across the participants and their experiences. We established a research committee to systematically analyze the four focus groups, aiming to identify saturation and emergent themes. Each member independently reviewed the transcripts as they coded responses and noted recurring patterns. Through discussions, themes were formed to assess similarities and differences ensuring consistency and reliability.

Key themes include the complexities of systematic bias, health disparities, the need for advocacy and empowerment, community outreach, and many more that emerged from the discussions further accentuating the challenges within the healthcare system. As a guide for evaluating the focus groups, critical questions led the analysis looking for parallels such as: Does the lack of being heard impact birthing experiences? What constitutes input in these scenarios, and what are the resulting outcomes? Is the quality of healthcare influenced by an individual's Black identity? Furthermore, does the presence of a diverse healthcare team lead to more positive outcomes for Black individuals under their care? The responses included in the study were selected based on relevance to the research objectives, clarity, and completeness. Each response was reviewed, and those that provided insights or directly addressed key discussion points were retained. To categorize responses as positive or negative, we assessed sentiment, tone, and content. Positive responses reflected agreement, satisfaction, or beneficial experiences, while negative responses conveyed dissatisfaction, challenges, or concerns.

In order to effectively organize and analyze our data, we paralleled a similar study that categorized perinatal experiences among women of color into individual, interpersonal, and macro/micro-group levels. This framework allowed researchers to organize interrelated concepts and identify levels of intervention to address emerging themes [6]. Adopting a similar approach, we structured our analysis across three theoretical levels: individual, interpersonal, and healthcare systems. This strategy enabled us to assess overarching concepts within each level and systematically code specific segments of qualitative data, providing a more organized and comprehensive understanding of participants' experiences.

## Results

Quantitative

**Figure 1:**
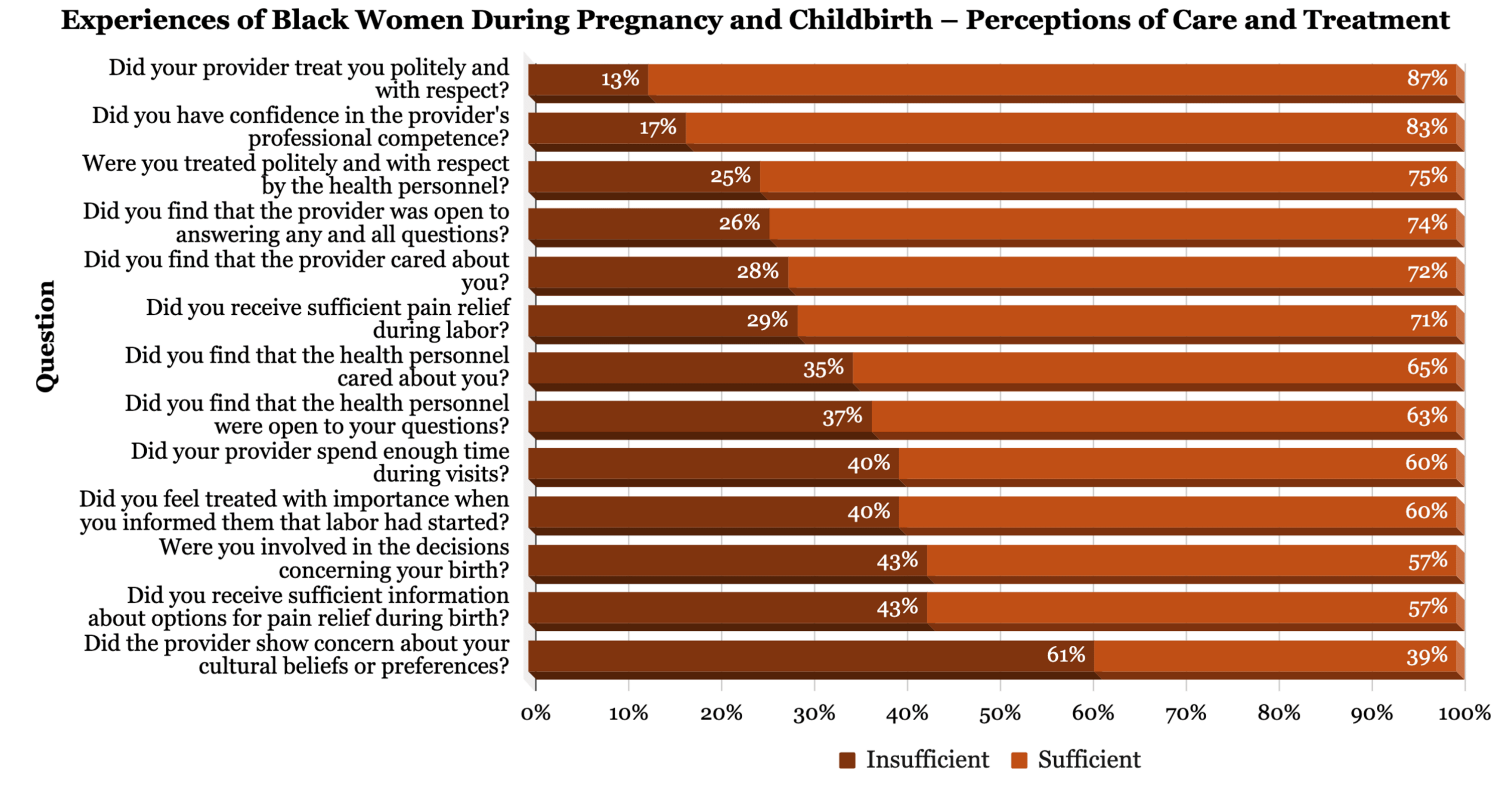
Bar graph of participants’ responses on quality of care, provider communication, respect, and cultural competence. Participants rated their experiences on a five-point scale, with scores of 4 or 5 classified as "sufficient," and scores of 3 or lower considered "insufficient," highlighting opportunities for improvement. Note: In order to format the graph, the wording of some questions have been condensed. The exact phrasing of the questions can be found in the appendix.

For the survey, the participant responses were divided into quantifiable and non-quantifiable results. The quantifiable results are those that were multiple choice and could be placed on a scale metric. To better understand participants’ perceptions of their care and treatment, we selected the questions that related most to this theme, which can be found in Figure 1. 

The final results of the survey consist of responses considered non-quantifiable. Throughout the questionnaire, there were sections included that allowed participants to expand on any thoughts, stories, or feelings they had surrounding a topic. To provide additional context, the free-response questions embedded in the survey can be found in the appendix, which offers insight into the specific prompts participants were answering. The following responses are negative accounts collected from the survey that Black women experienced in the healthcare system. 

Participant IDs that begin with A completed the questionnaire and focus group, while participant IDs that begin with B only completed only the questionnaire.

**(B-35):** *“*Last pregnancy was very complicated. A nurse compared me having babies to used baggage.*”*

**(A-10):**“I gave birth to both of my children while in the military. Options are limited because you are treated as GOVERNMENT PROPERTY.”

**(B-23):** “I had a White female nurse practitioner who was judgmental and rushed and took little care about me as a person. I had to advocate for another test during my pregnancy. They would discount my opinion due to my age.”

**(B-06):** “After first delivery, nurse (White female) in hospital told me to only use lotion on the baby because she knows Black people use other things. Fortunately another nurse (White female), told her that was inappropriate and questioned her statement to me. Third delivery, I had to repeatedly call for my child to be brought to my room. Even the day I was discharged I had to go to front desk after requesting my child be brought to my room twice, to show that we had been discharged.”

**(B-09):** “I wasn't listened to when I told them that I could feel the baby coming out. For the first birth they believed I couldn't feel anything b/c of the epidural, but it was a perfect epidural and I could feel the need to push and the baby coming out, even though I couldn't feel pain. The second time they had checked me recently, so they didn't believe it could be time. My (White) husband had to intervene both times to get them to check me.” 

**(B-11):** “I lost my baby after all, and the doctor kept saying it was a natural phenomenon because neither she, the doctor, nor I did anything wrong…” “With the exception of the one who died in [an] American hospital. I had the other three births in Africa, and I have them all in perfect condition.”

**(A-06):** “I had a terrible birth experience and I felt overall the providers I had did not care about my well-being. I tell women of color to avoid this hospital if possible. My birthing experience was horrific. I hope what happened to me never happens to another parent. I had to kick a nurse out of my room and told them if I saw her again, I would leave.”

These experiences highlight the need to ensure that healthcare workers and hospital systems are aware of how influential their comments and presence have on patients. 

On the contrary, individuals shared positive responses about their experiences as well as aspects that they valued and wished more providers would implement. These accounts can be found below. 

**(A-04):** “I always felt comfortable and heard during my appointments … Personable bedside manner and being able to explain things in an understandable way.” 

**(B-01):** “[The healthcare team] consistently checking in with me on pain level and making sure I was comfortable with my spouse by my bedside.”

In continuation with positive experiences, a shared thread emerged in the following accounts, revealing a meaningful aspect of participants' care. 

**(B-21):** “I went to female African-American providers. I truly believe it made all the difference in the world towards the care I received. I felt seen, heard, and safe.”

**(B-34):** “Having a Black OBGYN; being affirmed by my provider during and after giving birth.”

**(A-12):** “I had a Black/African American female OB/GYN, intentionally. She was very caring, thorough, and pregnant with me during my 1st pregnancy. We delivered days apart. She provided sound medical advice and took precautionary measures with me during pregnancy and thereafter for almost 20 years total. I was super sad when she departed from the practice, and continued to search for that type of service, comfort and confidence. My labor & delivery team were very attentive. I had scheduled induced labor for both pregnancies and remained pleased with that decision that my Dr., husband, & I made. Even during a miscarriage my Dr. provided optimal care and thoughtful advice. Care, confidence, and experience was consistent with my Dr and health team. In this instance, my Dr and I both were minorities and she was fully aware of the health disparities that often time affects minorities. She was a committed and passionate professional and I was blessed to be her patient for so many years. Even after birth she treated my anemia, fibroids, and provided mental health referrals to address changes throughout the years. I shared information and we worked as a team for the best treatment plans.”

**(B-36):** “My doctor was an African American woman and took every step to be attentive and educational. The only concern/issue was health care professionals continually questioning and doubting if that was my child because her complexion was so light at birth, compared to mine. It seemed as if they were not accustomed to the range of colors that African American children can be. Pressure to have a name for my child within 24 hours of her birth. My doctor truly cared about bringing my child into this world with the healthiest start to life, and would always envision the possible contributions to society each child could make. In order to realize her mission she had to provide the best care for mothers. As an African American woman with an African American doctor I do think my level of care was optimal and exceeded what I could have potentially experienced elsewhere. My OB/GYN has since retired, about four years ago. I have yet to find a replacement to have a proper/full exam. It’s so hard to find a quality and consistent care provider that meets my expectations/standards now. I’ve met two providers and each has rushed me through intro visits and failed to really care about my medical concerns through the lens of my personal health goals. Thank you for your research in this area!”

Qualitative

Participant Characteristics

For the qualitative analysis, we conducted five focus group interviews with a total of 14 participants, aiming for three to four women per group. The participants brought diverse and complex maternal health histories: two had experienced ectopic pregnancies, one was managing chronic hypertension, another had preeclampsia, and four had endured previous miscarriages. Some women navigated high-risk pregnancies, with one diagnosed with cervical cancer and another undergoing an emergency appendectomy during pregnancy. Delivery methods varied, with most participants delivering vaginally and five requiring cesarean sections.

In terms of demographic background, most participants identified as married, college-educated, and within the middle range of socioeconomic statuses, holding professions such as teachers, healthcare workers, government employees, military personnel, an attorney, and more. All participants had medical insurance, yet despite this relative privilege, their experiences revealed significant disparities and challenges within the healthcare system. Participants offered invaluable insights into their unique encounters with pregnancy and childbirth by communicating their stories through Zoom.

Qualitative Findings

From the focus groups, qualitative data were organized and analyzed into three theoretical levels: individual, interpersonal, and healthcare system. Table 1 presents the overarching themes that emerged from each level, which emphasizes key issues contributing to the ongoing disparities in maternal healthcare among Black/African American women.

**Table 1:** Key themes identified with associated codes.

Theoretical levels	Themes	Codes
Individual	Lack of knowledge; increased necessity to self-advocate	Lack of tangible health information
Interpersonal	Interaction with providers	Lack of urgency from providers; feeling marginalized; feeling unheard
	Support systems	Prenatal and postnatal programs and services; family members, friends, partners, others, etc.
Healthcare system	Access and availability	Access barriers (transportation, insurance, etc.)
	Communication and information	Provider-patient communication
	Equity and discrimination	Patient education and empowerment
	Cultural competency	Cultural competency training for healthcare providers

The table captures the broad spectrum of maternal experiences and their associated outcomes. These themes illustrate the complex realities of maternal healthcare for Black/African American women, highlighting critical experiences that shape the problems within each theoretical level.

**Individual level**: At the individual level, many new or first-time mothers have expressed deep-rooted anxieties about navigating the unknowns of pregnancy. This lack of knowledge spanned multiple areas, including physical and emotional changes, understanding the birthing process, and feeling adequately prepared to care for a newborn. Participants shared personal anecdotes that vividly illustrated their uncertainties and emotional struggles. The following accounts capture two mothers' experiences: one reflecting on the challenges of giving birth at a young age, grappling with the decision to request pain medication, and another describing the physical transformations of pregnancy and the impact on her comfort and well-being.

**(A-02):** “They just didn't offer you anything. I mean, it was my first child. So I didn't really know to ask for anything. And they didn't say like, do you want anything for pain? And, you know, I guess I just start thinking, this is just what it is. I was only like 20, 22, or 23. So, you know, I just, I just bared it.”

**(A-06): **“Pregnancy was really uncomfortable, but I think that's just an aspect of pregnancy. I was an athlete in high school and college, and so I was really used to being comfortable in my body, and being pregnant was not that. So I felt really uncomfortable.”

At the individual level, many pregnant women expressed difficulties with self-advocacy regarding their health. This struggle often stemmed from anxiety surrounding the doctor-patient relationship, limited knowledge about pregnancy, or uncertainty in recognizing abnormal symptoms. When healthcare professionals dismissed or disregarded women’s concerns, it heightened the necessity for patients to advocate for themselves, which can feel like a burden that should not rest solely on the individual.

Participants recounted the benefit and importance of having someone who can advocate on your behalf at every point of pregnancy, especially the birth. This is an essential facet that can make an immense, positive impact on a patient’s healthcare journey: 

**(A-09):** “My best friend that was with me has a medical background so she would ask a lot of questions. To this day, when I go for follow-up, one of my doctors says, “So how's your scary friend?” He categorizes her as scary because she was advocating. She put her foot down and she was asking questions.”

**(A-14):** “My takeaway to new mothers is to always have a female advocate who's given birth before and will move heaven and earth to make sure you get what you need. Also, be sure it's someone who is aware of how things should go.”

Some focus group participants mentioned local programs that assisted them after birth. However, these programs are not widely known, and education would facilitate and increase the utilization of such programs and their benefits for women.

**(A-04):** “And then they also have like services through insurance like if you happen to experience like any postpartum or anything like that. They have like the EAP [Employee Assistant Programs] where you can reach out to folks if you need any help.”

Addressing the knowledge gap is crucial, particularly for Black pregnant women. With interventions aimed at providing comprehensive education on the period of transformations during pregnancy and the array of birthing options available, we can empower expectant mothers to make informed decisions about their healthcare and improve maternal health outcomes within this community.

**Interpersonal level**: Participants shared experiences of feeling disregarded, disbelieved, and marginalized by their providers. The lack of rapport between healthcare professionals and Black pregnant women deepens the disconnect between patients and the healthcare system. Many women recounted moments where their desires were not heard, and their power in decision-making was hindered, leading to feelings of frustration and emotional distress. These communication challenges often shaped participants' negative perceptions of their hospital visits, reinforcing the notion that their well-being was secondary to systemic biases.

**(A-06):** “She didn't listen. She was the second person, I said, I don't want to see your face again. Because she just wasn't listening. And I was so frustrated. It's been 24 hours with up and down pain cycles and very low communication about what the plan was.”

**(A-08):** “White nurse and she just pulls my IV out and I said, well, I'm still having some pain and my pain… it's like an eight because I've been trying to stretch the pain medication. She's like, well, if you're in that much pain then I guess you just need to stay another night and you can have a conversation with your doctor, but I'm bringing you Tylenol. So do you want Tylenol or not? And I'm like, wow.”

**(A-05):** “I guess I just wasn't believed. I don't think I had regular contractions, but nobody wanted to believe me.” 

People often underestimate the power of choice in healthcare. While factors like insurance coverage and limited access can make switching providers difficult, it is essential to prioritize feeling valued and understood. If circumstances allow, individuals should not hesitate to seek a different provider when they feel disconnected or overlooked as one participant (A-08) noted: “If a provider doesn't work for you, don't hesitate to switch that provider."

This gap between patients and the healthcare system widens when negative experiences accumulate, fostering mistrust and reinforcing systemic inequities. The stories shared by participants illustrate the pervasive challenges Black women face during pregnancy and childbirth, underscoring the urgent need for reform. Addressing issues such as implicit bias, community care gaps, and the absence of patient-centered care is crucial to rebuilding trust and improving maternal health outcomes. Amplifying Black women’s voices is essential not only for fostering understanding and empathy but also for catalyzing change among healthcare providers, policymakers, and the broader community.

While many participants described feeling dismissed by physicians, some also highlighted the profound impact of midwives and nurses who showed compassion and intentionality in prioritizing patient desires. These healthcare team members often became essential advocates, bridging the gap between patients and physicians to ensure their voices were heard.

**(B-09): **“Midwives show a lot more care towards their patients than the doctors do in my experience.” **(B-06): **“I had really good nurses who I provided my birthing plan to, and they were my biggest advocates, even challenging the doctors.”

These accounts emphasize the importance of trust and support within the care team, especially when patients feel unheard by their physicians. However, the inconsistent presence of culturally competent care across all team members remains a challenge that leaves patients vulnerable to biases and communication breakdowns that persist within the healthcare system.

**Healthcare system level**: At the healthcare system level, multiple structural barriers remain evident, such as access and availability, which can be the result of transportation issues, insurance limitations, and the scarcity of culturally competent providers. In addition, breakdowns in communication further exacerbate these challenges, leaving many Black women feeling misunderstood and overlooked. One participant described the bias and marginalization she experienced, noting a clear disparity that had been instilled in healthcare providers historically, and some may still unconsciously believe today.

**(A-06):** “You know, you're still dealing with the bias. The issue is the kind of baseline care. And that to me is really scary that we as a society have decided that's appropriate. But it is kind of that baseline care. And you know, I definitely feel like a lot of that is driven by bias in nursing. And then I do feel like it's also the healthcare providers not believing black people experience pain.”

Biases within healthcare settings hinder equitable treatment, making true healthcare equity elusive. Cultural competency among healthcare professionals remains inconsistent, often leading to misinterpretations of symptoms, dismissal of concerns, and inappropriate care. Efforts to bridge these gaps must extend beyond improving access; they must also emphasize respectful, empowering provider-patient communication. This includes comprehensive patient education initiatives and mandatory cultural competency training for providers to ensure Black women receive equitable, culturally sensitive care that honors their autonomy and dignity.

Throughout the stages of pregnancy, the provider role is essential in guiding the patient through medical decisions and helping them understand bodily changes. This support becomes even more impactful when patients feel represented and understood by their provider. One participant (A-07) shared: “A Black doctor, a Black female doctor, has made a difference in my experience, you know, and it sounds like that has been the other ladies’ experiences also. I think it’s their perspective and the background knowledge they bring to their patients. Not only is the patient advocating, but they have a doctor advocating for them, and that makes a difference.” This sentiment powerfully underscores how provider representation, advocacy, and cultural understanding can shape maternal health experiences. When patients feel seen and valued, it alleviates the need for constant self-advocacy and allows them to navigate their pregnancy journey with greater confidence and trust in their care.

**Figure 2:**
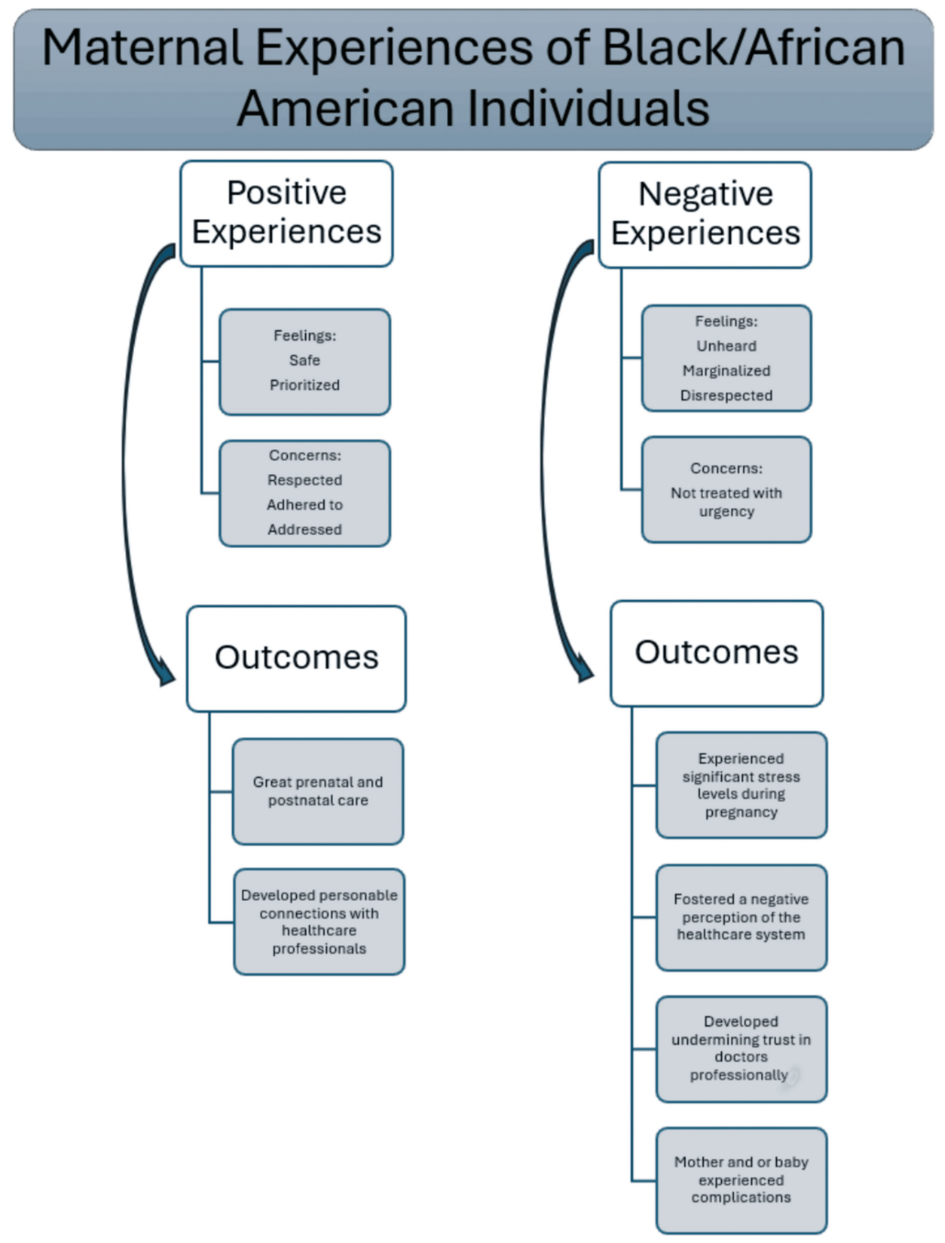
Maternal experiences of Black/African American individuals and their associated outcomes. A wide range of experiences and overarching themes were categorized into positive and negative experiences and outcomes from participants within the healthcare system.

The experiences of Black women during childbirth reveal a profound connection between the quality of care they receive and the outcomes they experience. To illustrate these dynamics, we have developed a figure that organizes these experiences and their corresponding outcomes (Figure 2). This framework underscores the critical importance of equitable and respectful maternal care in ensuring positive birth experiences and health outcomes for Black women.

Ultimately, the preceding model urges the future development of a comprehensive program that entails interventions to improve patient advocacy as well as educational initiatives to empower women with knowledge about their bodies and their power to demand appropriate care. From our study, participants suggested changes that should be implemented in our systems to prevent negative experiences and improve the healthcare system. 

**(A-03):** “It's just trying to get the education out there. It's putting the education in the clinics. It's putting the education in the high schools, even in the colleges, you know, in the going into the community centers, the welfare office, and the project.” 

**(A-02):** "I was trying to say that we have to empower the patient to have enough self-esteem in their selves and to care about themselves enough to fight for themselves on what they deserve and what they are going to want."

**(A-14):** “I think I have more to say to the young women than to the doctors, other than telling the doctors that were litigious. So they do the right thing upfront. But I think that as a culture, us understanding that we're paying these doctors, these doctors work for us. And if we don't like the care we're given and we have the option to choose someone else, to choose someone else. And I think that we have to prepare for these appointments. Our life and our children's lives are depending on it.”

With more education implemented into schools and medical systems, every individual can be equipped with more tools to navigate healthcare as well as an increase in the knowledge and skills to create nurturing environments that can lead to improved outcomes and patient experiences. 

Highlighting Comprehensive Accounts

The focus group interviews gave an invaluable experience of understanding the stories and experiences of many Black American women during pregnancy. With insight into their anecdotes, the challenges were highlighted, which include facing dismissive behavior from healthcare providers, lack of culturally competent care, ineffective communication, and more. The personal accounts shared by individuals all add meaning and dialogue that leads to understanding why there is such a crucial need for systematic changes within healthcare. Out of the vast experiences we analyzed, we wanted to highlight two stories that revealed immense issues within the quality and urgency of care, especially for Black women. By amplifying their stories, we aim to spark change that will address these concerns so that other women do not have to undergo the same negative, and often traumatic, experiences.

**Story 1 (A-05)**: As contractions intensified, she knew it was time. This was not her first time; she was about to give birth to her fifth child. But like her previous experiences, her contractions didn't follow the usual pattern. Instead of the expected progression, they were irregular, coming every 10 minutes and then suddenly escalating into the intensity of labor. She and her husband rushed to the hospital, hoping for a smooth delivery. However, upon arrival, the attending doctor seemed disinterested and indifferent to her unique situation. Despite her explanation of irregular contractions and the urgency of the moment, the doctor decided to send her home.

Frustrated and bewildered, she found herself back in the familiar comfort of her home. But as the minutes progressed, the intensity of her contractions intensified, further signaling that she was ready to deliver, whether a doctor was present or not. Amid the chaos, she braced herself for the inevitable with her husband delivering the baby in their living room. It was not the serene hospital environment she had envisioned, but in that moment, as she held her newborn in her arms, she felt a surge of empowerment and resilience.

Meanwhile, at the hospital, verbal accounts of the doctor's negligence spread like wildfire among the medical staff. The other doctors were livid with their colleague's disregard for her well-being.

It later emerged that the doctor wanted her to return to the hospital where he worked, not out of concern for her safety but to ensure he could still bill for the delivery despite having played no role in it. His actions, or lack thereof, were unacceptable, especially considering he did not bother to check on her before her discharge.

**(A-05):** “They sent me home. His other doctors were so livid with him. He actually wanted me to come back to the hospital where he worked so he could still bill even though he had nothing to do with the delivery. He sent me home without even coming to check on me before me being released when he knew that I have irregular birth. Like my contractions don't go the normal, you know, five minutes, and then again …. I don't follow a pattern. I go from my contractions for 10 minutes, then giving birth. Like that. He knew that and he would not come check on me and let them send me home.”

**Story 2 (A-14)**: During labor, the medical team broke her water and discovered meconium staining, a sign that indicates that the baby had passed stool before birth, a critical indicator of distress. However, the information was not communicated to her or her husband. She distinctly remembered the unpleasant smell associated with the meconium stain but was unaware of its significance.

Meanwhile, her baby was stuck in the birth canal, leading the medical team to declare an emergency C-section because of concerns of low oxygen levels. However, this supposed emergency procedure took half an hour to begin questioning the urgency of the situation.

After the birth of her newborn, the baby was diagnosed with jaundice and placed in the NICU. He was later diagnosed to be on the autism spectrum around the age of 2-3, which his mother believes could be a result of the low oxygen levels during his birth.

Efforts to obtain medical records posed a new set of challenges for the couple as hospitals lost medical records. After retrieving the records, they discovered information that needed to be communicated with the couple but was not. This communicates that there is often a disconnect between patients and the medical team, which affects the quality of care.

**(A-14):** “It's really hard to advocate for yourself when you are in that state and it's the first time. My husband, bless his heart, didn't have a clue what to expect either. We tried our best.”

**(A-14):** “I advise everybody that the husband gets somebody who knows what's supposed to happen who can advocate for you. And there became a point where I think he (the baby) just was stuck in the canal. First of all, my, they had to break my water. And they saw meconium staining. They didn't tell us what it was. We remember the experience because meconium staining has a certain scent to it. It doesn't smell good. And we remembered that. But no one said to us what had happened. He wasn't coming down the canal. And they said he was low on oxygen. So they were going to do an emergency C-section. But the emergency C-section took like 30 minutes. I remember thinking, how is that an emergency? And then, I know if I had a parent in the room, they would have, you know, all hell would have broken loose. But as young parents, we were, you know, like you guys, trying not to make too much of a fuss. And so when he was born, no one knew we didn't know he was on this spectrum for probably two and a half, three years. And then later on, I was doing research for a book. And I tried to get the records and Kaiser Permanente had lost them, but Fairfax Hospital where he was born had them. And there was information in the records that they never told us about. Like they never told us about the Meconium staining, they never did and I also had a fever going in. He was Jaundice, he was in the NICU. And he wasn't a small baby. And I did get to like ... got to stay the whole time he was there. But all those things, I think were factors to his diagnosis later on.” 

## Discussion

The findings from both components of this study highlight the complex and deeply personal experiences of Black women in maternal healthcare, reinforcing the persistent disparities that permeate medical settings. According to the CDC, maternal mortality rates have declined for White and Hispanic women. However, the rate for Black women has increased to 50.3 deaths per 100,000 live births, which is a figure that remains more than three times higher than the rate for White women (14.5) and over four times higher than the rate for Hispanic women (12.4) [7]. These staggering statistics lay bare the severe and ongoing racial inequities in maternal health outcomes.

Critical and intersectional scholars argue that these disparities are not anomalies but rather evidence of systematic mistreatment and neglect within the healthcare system [8]. Black mothers are more likely to experience postpartum separation from their infants, inconsistent breastfeeding education, and inadequate follow-up care for conditions such as high blood pressure and cervical complications [3]. These failures in care contribute to poor health outcomes and erode patient trust in the system.

However, statistics alone cannot capture the full scope of these disparities. By examining the lived experiences of Black women navigating pregnancy and childbirth, this study reveals two overarching themes: negative encounters marked by bias, dismissal, and inadequate care, and positive experiences shaped by provider attentiveness, cultural competence, and trust. These findings align with prior research, emphasizing the profound impact of racial bias in maternal healthcare and the critical importance of representation in fostering safer, more supportive patient-provider relationships [9,10].

Dismissal and bias

The negative accounts shared by participants illustrate recurring challenges faced by Black women in hospital settings. Many respondents described instances of their concerns being dismissed, particularly during labor and delivery. For example, one participant (B-09) detailed how her providers refused to believe she was ready to push despite her insistence, only responding when her white husband intervened. This aligns with an article indicating that Black patients' pain and concerns are more likely to be underestimated by healthcare providers, leading to delays in necessary medical interventions [11]. To further emphasize this issue, another participant’s experience (A-05) was particularly striking. Despite arriving at the hospital to give birth to her fifth child, the healthcare team refused to acknowledge her understanding of her own body. Due to their lack of urgency and care, she was sent home, leading to a critical situation where she ultimately gave birth in her living room. This key finding in our study is the recurring theme of dismissal and marginalization experienced by Black women in clinical settings. Based on personal experiences, participants reported instances where their concerns were minimized, their symptoms were not taken seriously, and their voices were overlooked in medical decision-making. These accounts align with existing literature on racial disparities in healthcare and reinforce the necessity of patient self-advocacy. Mothers receive less monitoring, their concerns are frequently dismissed, and they are often discharged without sufficient information about potential warning signs. For African American mothers, the risks increase at every stage of labor, delivery, and postpartum care [12]. 

Additionally, several responses reflected racial bias in provider-patient interactions, including inappropriate comments and assumptions about cultural practices. One participant (B-06) recalled being told to only use lotion on her baby “because Black people use other things,” a microaggression that exemplifies the lack of cultural awareness among some healthcare workers. Research has shown that implicit bias contributes to Black women receiving lower-quality care and less patient-centered communication [13]. Race-related attitudes among physicians, even if implicit, influence the quality of communication in patient-physician interactions and thus impact the disparities in treatment and information exchange [14]. Such interactions not only affect patient experiences but can also influence medical outcomes by discouraging patients from seeking care in the future.

Supportive healthcare

Despite many distressing experiences, some participants also shared stories of compassionate, patient-centered care that highlight the profound impact of culturally competent providers and supportive healthcare environments. One participant (A-12) expressed how her Black female OB/GYN provided attentive, thorough care and remained a trusted medical resource for nearly 20 years. Another respondent (B-21) directly attributed their positive experience to having an African American provider, stating they felt “seen, heard, and safe.” These findings align with research showing that Black babies are more likely to survive childbirth when cared for by Black doctors and three times more likely to die when cared for by White doctors [7]. This study, along with others, suggests that racial concordance between patients and providers can lead to improved health outcomes due to increased trust, improved communication with a greater sense of being understood, and a higher likelihood of patient-centered care [14]. When Black women are asked about how to improve perinatal care, they consistently request Black health providers [15]. The results of this study further support the need for increased diversity in the healthcare workforce, expanding access to Black providers, and the importance of cultural competence training for all providers in order to address these disparities. 

To build upon positive experiences, one recurring theme was supportive environments through family and community networks which functioned as sources of encouragement, a means of advocacy when navigating with the healthcare team, and a sense of comfort. As one participant (A-08) noted, "And if I didn't have a church family, it would have been even worse for me if I didn't have a good support system." This highlights the need for prenatal care models that intentionally integrate and strengthen community connections. One concept that can bolster the importance of community is the idea of group prenatal care, which is a model where pregnant women with a similar due date attend their appointments together, which fundamentally alters service delivery by providing psychosocial support which can be vital for individuals who may be enduring their pregnancy in isolated conditions [16]. The concept of group healthcare disrupts traditional power hierarchies, promotes interactive learning, and builds vital social support networks by fostering a sense of community [15]. Patients who participate in group healthcare feel less alone in their journey, as the presence of peers creates a supportive environment where they can openly share experiences, advocate for one another, and engage in collaborative learning, shifting the traditional provider-patient power dynamic. Group care models provide not only medical support but also a sense of belonging, reducing stress, and improving health outcomes [15]. Additional benefits of group care include higher prenatal and postnatal care satisfaction, improved mental and physical health, better breastfeeding behaviors, and increased contraceptive uptake to support healthier birth spacing [15]. Moreover, many are looking into alternative ways to give birth other than the hospital. Black doulas and midwives play a crucial role in improving maternal and infant health outcomes within Black communities and are increasingly being incorporated into birth programs [17]. By integrating community into one’s healthcare, positive outcomes can certainly be enhanced. Midwives, doulas, and group care provide essential support by offering competent care, reducing disparities in maternal health outcomes, and fostering a strong support system that empowers Black mothers through advocacy, education and emotional guidance.

Education and advocacy

A multifaceted approach is essential to addressing disparities in maternal healthcare. Comprehensive reproductive health education should begin as early as high school, equipping young women with foundational knowledge about their bodies and healthcare rights. A participant (A-02) urged the need “to educate the young girls more about themselves and their bodies and so that they have more pride in themselves and care for themselves.” Equipping Black women with healthcare information is crucial for strengthening patient advocacy, improving health outcomes, and addressing disparities in medical care. Black women face unique challenges in the healthcare system, including implicit bias, higher maternal mortality rates, and inadequate pain management [9]. For instance, accessible pamphlets and resources should be made available in clinics and community centers to ensure women have the information they need to navigate the healthcare system effectively. Furthermore, central to these efforts is the promotion of self-advocacy, enabling women to ensure their voices are heard and their concerns addressed as active participants in decisions about their health. Providing patients with knowledge about their rights, treatment options, and available resources empowers them to make informed decisions, advocate for equitable care, and challenge systemic barriers [18]. Patients should also know of their right to choose the provider that is compatible with them and would adhere to their concerns. Patients play a crucial role in achieving optimal health by actively participating in treatment decisions and seeking alternative physicians if their care is unsatisfactory [19]. One participant (A-08) highlighted this: “If a provider doesn't work for you, don't hesitate to switch that provider." Additionally, studies have shown that when patients are well-informed, it enhances engagement in shared decision-making with their providers, leading to improved health outcomes and increased trust in the medical system [20]. By enhancing patient education and advocacy, healthcare systems can help Black women navigate medical spaces more effectively and ensure they receive high-quality, unbiased care. 

Although individual action is vital, in our view, the most critical factor is that healthcare providers must commit to identifying and addressing their own biases while continuously strengthening patient-centered communication skills. By establishing a connection with clinicians and facilitating clear communication, patients feel more at ease [21]. Patients should always be listened to, and their concerns should be acknowledged and addressed with the utmost care and thoroughness. Active listening and learning how to provide the best care are not innate qualities-they are skills that must be intentionally taught, nurtured, and reinforced, not just in medical school but throughout a physician’s career as part of lifelong learning. Research highlights that cultural competence is a multifaceted skill set involving awareness, knowledge, encounters, and a genuine desire to understand diverse patient experiences. The study emphasizes that while individual healthcare providers play a crucial role, systemic constraints within healthcare institutions often hinder the development and implementation of cultural competence. To address this, collaborative efforts between healthcare organizations, policymakers, and educators are necessary to improve provider training and ultimately enhance patient care [22]. Integrating such training into both medical education and continuing professional development can equip healthcare providers with the tools necessary to recognize implicit biases, engage in meaningful self-reflection, and develop strategies to mitigate their impact on patient care. The implementation of empathetic medical education will foster more compassionate, equitable care and build stronger, trust-based connections between providers and patients. Ultimately, a healthcare system rooted in empathy, respect, and cultural awareness will not only improve patient outcomes but also promote a more just and inclusive environment for all.

It is essential to ensure the specific needs and experiences of the Black community are met, which can happen by building trust, connection, and dialogue that foster supportive and informed environments. Studies show that Black patients, when given a choice, often prefer physicians who share their racial or ethnic background [9,23]. This preference is reflected in our study, where many women attributed their positive pregnancy experiences to being cared for by Black doctors, highlighting the critical role of representation and advocacy in patient care. While all physicians, regardless of race or ethnicity, should be committed to fostering trust and providing equitable care, these findings reinforce the importance of diversity in the healthcare workforce. Many of the positive experiences shared by respondents directly correlated with receiving care from Black or culturally competent providers, suggesting that increasing diversity and implementing structured cultural competence training could be key strategies in reducing disparities and improving maternal health outcomes.

Action and reform

To translate these insights into meaningful change, healthcare systems must commit to concrete, systemic improvements. First, mandating cultural competency training for all healthcare providers is essential. This training should extend beyond surface-level awareness to include bias recognition, patient-centered communication techniques, and historical context on healthcare disparities. Continuous education, rather than one-time courses, will help ensure long-term improvements in provider-patient interactions. Furthermore, expanding community-based care models can bridge gaps in accessibility and trust. By investing in Black-led maternal health initiatives, midwifery programs, and community health workers, culturally congruent care and advocacy can be provided along with patients receiving support from professionals who share commonalities and wisdom surrounding their lived experiences as people of color. Additionally, enhancing patient education and advocacy resources within clinical settings can empower all women to navigate the healthcare system effectively. Clinics should provide accessible, plain-language materials outlining patient rights, provider selection strategies, and self-advocacy techniques. Finally, prioritizing workforce diversity is critical. Recruiting and retaining more Black physicians, midwives, and doulas will help create an environment where patients feel seen, heard, and respected. Structural policies that support career pipelines for underrepresented medical professionals can lead to a more inclusive and equitable healthcare landscape. By implementing these changes, healthcare systems can move from passive acknowledgment of disparities to active solutions that drive measurable improvements in maternal health outcomes.

Study limitations

A limitation of our study is the relatively low sample size, which may reduce the generalizability of our findings to the broader population. Additionally, while 60 participants completed the survey, only 14 opted to participate in follow-up discussions within the focus groups. This self-selection may introduce bias, as those who experienced more negative maternal healthcare encounters may have been more inclined to share their stories. As a result, our qualitative findings may not fully represent the diversity of experiences within the Black maternal health community. Furthermore, another limitation of this study is that most participants were college-educated and based in Virginia, which may not fully capture the diverse experiences across different socioeconomic, educational, and geographic backgrounds. Nonetheless, despite their educational attainment, the voices and experiences shared by participants still reported significant widespread challenges in maternal healthcare, which highlights that these issues persist across the socioeconomic spectrum. However, this raises concerns that individuals with lower levels of education or from lower socioeconomic backgrounds may be experiencing even greater hardships in maternal healthcare. This reality underscores the urgency and importance of addressing these disparities and implementing policy and practice reforms that ensure equitable maternal care for all.

Future direction

Future research should seek to include a more diverse participant pool as well as a more extended time period of data collection to gain deeper insight into how socioeconomic status, education level, and geographic location influence the healthcare experiences of Black women. By expanding the range of participants, there will be a more comprehensive understanding of systemic disparities, which further reinforces efforts to advance equitable, patient-centered care.

## Conclusions

The findings from this study underscore the urgent need for comprehensive reforms to address disparities in maternal healthcare among Black/African American women. By centering the personal narratives of Black women, this research moves beyond statistics to humanize the lived experiences behind the data and offer a patient-centered perspective that is often overlooked in discussions of maternal health disparities. Addressing systemic biases, empowering patients and communities, and ensuring equitable access to high-quality maternal care are essential steps toward creating a more inclusive and effective healthcare system. Looking forward, interventions should focus on bridging knowledge gaps, enhancing patient advocacy, and providing comprehensive clinician training. These measures will aim to create inclusive, accessible, and sustainable solutions that will strive towards a more equitable healthcare system that addresses the needs of those most disproportionately affected by maternal mortality across our nation. The ultimate objective of this research is to save lives, reduce healthcare disparities, and improve patient experiences across our nation.

## Appendices

**Table 2:** Survey questionnaire. Note: Question #3 refers to the section where we share information to participants regarding who they can contact if they have any questions regarding our study or their rights as a research subject.

Block	Question number(s)	Questions
Introduction	1–4	1. Background + purpose
		2. Consent approval
		3. Questions
		4. We are seeking experiences from Black/African American individuals. If you classify as such, please select your racial identity below
Pregnancy	5–31	5. Did you use standard health services during pregnancy (i.e. check-ups or routine ultrasound scans)?
		6. From whom did you receive your check-ups during pregnancy? (Select all that apply)
		7. How many weeks into pregnancy were you when you had the first pregnancy checkup by a midwife or doctor?
		8. How many pregnancy checkups did you have in total from a midwife/doctor?
		9. Do you think this was an appropriate number of check-ups?
		10. Was it easy to get an appointment with your provider if you wanted to?
		11. How many providers did you see during your pregnancy?
		12. Did your provider treat you politely and with respect?
		13. Did your provider spend enough time during visits?
		14. Did you find that the provider was open to answering any and all questions?
		15. Did you find that the provider cared about you?
		16. Did you have confidence in the provider's professional competence?
		17. Did you receive sufficient information about your physical health during the pregnancy?
		18. Did you receive sufficient information about possible mood changes during the pregnancy?
		19. Did you receive sufficient information about how the baby was developing?
		20. Did you receive sufficient information about what you could expect regarding birth?
		21. Did you receive sufficient information about options for pain relief during birth?
		22. Did you receive sufficient information about post-natal period (i.e. breastfeeding, nutrition, care for the child)?
		23. Did you ever feel that health personnel gave you conflicting information?
		24. How many ultrasound scans did you have?
		25. Did you think this was an appropriate number of ultrasounds?
		26. Did you receive sufficient information concerning the ultrasounds?
		27. Did you find that the services you received were well organized?
		28. All in all, were the services you used during pregnancy satisfactory?
		29. All in all, were the services you used during pregnancy what you expected?
		30. Did the provider show concern about your cultural beliefs or preferences?
		31. Did you notice a negative influence on your mental health and wellbeing due to the care you received during your pregnancy?
The Birth	32–36	32. Where did you give birth?
		33. Did you give birth to one or more children?
		34. How many weeks into the pregnancy were you when the child/children born?
		35. What was the child's birthweight? If you gave birth to twins or more, please answer according to the child that was born first.
		36. How did you give birth?
The Stay	37–53	37. Did you feel that you were treated with importance by the health personnel at the delivery ward when you informed them that labor had started?
		38. Were you treated politely and with respect by the health personnel at the delivery ward?
		39. Did you find that the health personnel were open to your questions?
		40. Did you find that the health personnel cared about you?
		41. Did you have confidence in the health personnel's professional competence?
		42. Did you receive sufficient information during your stay at the delivery ward?
		43. Did you experience that health personnel gave you conflicting information?
		44. Did you find that the services you received during your stay at the delivery ward were well-organized?
		45. Did you find that the health personnel cooperated well during the birth?
		46. Were you involved in the decisions concerning your birth?
		47. Did you receive sufficient pain relief during labor?
		48. All in all, were the services you received during your stay satisfactory?
		49. All in all, were the services you received during your stay what you expected?
		50. Are you of the opinion that your child was in any way given incorrect treatment (according to your own judgement)?
		51. Are you of the opinion that you were in any way given incorrect treatment (according to your own judgement)?
		52. Did you have a consultation with a provider before going home?
		53. What benefit did you have from this consultation?
Background	54–59	54. What is your age?
		55. Are you married or cohabiting?
		56. What is your highest completed level of education?
		57. What is your everyday activity when you are not on maternity leave?
		58. Overall, would you say your health is ...
		59. How many times have you given birth in the past?
Free Response Questions	60–65	60. Are there any aspects of any birth that you’ve experienced that you believe should have never happened?
		61. Are there any aspects of any birth that you’ve experienced that you valued and wish more providers implemented?
		62. Are there any aspects of your pregnancy or birthing experience that you feel could’ve been attributed to your racial identity?
		63. Was there any point where you felt that you had to advocate for yourself to receive care that you were not initially given?
		64. If there is anything else you’d like to share, please feel more than welcome to do so below:
Invitation for Interview Groups		65. We will be conducting focused interviews with Black/African American individuals who would like to dive deeper into their birthing experiences to contribute to our research. If you’d consider participating, we would love to talk more and we’d appreciate it if you'd list your information below. We will be selecting multiple participants to receive a $50 gift card upon completion. Thank you so much!

**Table 3:** Interview guide for focus groups.

Category	Questions	Probing Indicators
Opening + clinical factors	1. How many times have you been pregnant?	Include miscarriages, abortions, live births, number of living children
	2. Did you have any prior health concerns before pregnancy?	Probe for physical health, weight, mental health, reproductive health
	3. Were you taking any medications before, during, or after pregnancy?	Ask about prescriptions, over-the-counter medicines, vitamins, supplements
	4. What can you tell me about the healthcare you received while pregnant?	How was the provider selected? Type of provider? Frequency of visits? Continuity of care?
	5. How did you feel about the care you received from your doctor, midwife, or nurse during pregnancy?	Did they listen to concerns? Were they respectful? Did you feel comfortable with them?
	6. Did you have any physical complications during pregnancy?	Probe for high blood pressure, seizures, diabetes, blood clots, bleeding, depression/anxiety, C-section recovery, infection, severe tears, NICU, stillbirth
	7. Were you referred to any other doctors, clinics, or hospitals?	Were you able to visit the referred provider? If not, why? What happened during the visit?
	8. Can you describe the healthcare you received after giving birth?	Did you attend postpartum checkups? Any challenges in accessing care? Any complications?
	9. How did you feel about the postpartum care you received?	Did the provider listen to concerns? Did you feel comfortable and confident in the care?
	10. Were there times you were seriously concerned about your pregnancy or health?	What were the concerns? How were they addressed? Who provided information and care?
	11. What do you think were the events leading to those concerns?	Probe for information sources, communication clarity, involvement in decision-making
Social determinants of health factors	12. Did you worry about money or health insurance during or after pregnancy?	What financial issues arose? How did they affect your healthcare access?
	13. How did you feel about where you lived during or after pregnancy?	Probe for housing stability, neighborhood safety, access to services, distance to work
	14. What kind of support did you have during or after pregnancy?	Probe for food, housing, transportation, medical care, financial assistance, emotional support
	15. Did you experience anxiety, depression, or mental health challenges?	How were you coping? Do you think your feelings were linked to healthcare experiences?
	16. Were you seeing a provider or counselor for emotional/mental health?	What was the experience like? Was treatment accessible and helpful?
	17. How did your emotional or mental health affect your relationships?	Probe for impact on family, partner, work, and social interactions
	18. Did you use substances (prescription narcotics, alcohol, tobacco, drugs) during or after pregnancy?	If yes, how did it affect daily life and childcare? Did you seek treatment?
	19. How would you describe your relationship with the baby’s father?	Clarify relationship type (spouse, partner, co-parent). Probe for emotional support and involvement
	20. Were you abused or neglected as a child?	What can you share about those experiences? Did you receive counseling or support?
	21. Is there anything else about your life or health experiences you’d like to share?	Open-ended prompt for additional insights
Closing questions	22. What do you think could be done to better support women during pregnancy and childbirth?	What advice would you give to healthcare providers?
	23. Would you like a list of resources that may be helpful?	How would you like to receive the list (mail/email)?
